# Supplementary material for: A time-resolved multi-omics atlas of Acanthamoeba castellanii encystment
Source: Nat Commun. 2022 Jul 14;13:4104. doi: 10.1038/s41467-022-31832-0 (PMC9283445; doi:10.1038/s41467-022-31832-0)
Supplement: Supplementary file 1 — Supplementary Information [file 41467_2022_31832_MOESM1_ESM.pdf]

Supplementary Information

A time-resolved multi-omics atlas of *Acanthamoeba castellanii* encystment

Clément Bernard[1]*, Marie Locard-Paulet[2]*, Cyril Noel[3]*, Magalie Duchateau[4], Quentin Giai Gianetto[4,5], Bouziane Moumen[1], Thomas Rattei[6], Yann Hechard[1], Lars Juhl Jensen[2], Mariette Matondo[4] and Ascel Samba-Louaka[1]✉

[1] Laboratoire Ecologie et Biologie des Interactions, Université de Poitiers, UMR CNRS 7267, Poitiers, France.

[2] Novo Nordisk Foundation Center for Protein Research, University of Copenhagen, Denmark.

[3] IFREMER-IRSI-Service de Bioinformatique (SeBiMER), Centre Bretagne, Plouzane, France.

[4] Institut Pasteur, Université de Paris, Proteomics Platform, Mass Spectrometry for Biology Unit, UAR2024 CNRS 2000, Paris, France

[5] Institut Pasteur, Université de Paris, Department of Computation Biology, Bioinformatics and Biostatistics Hub, Paris, France

[6] Centre for Microbiology and Environmental Systems Science; Doctoral School Microbiology and Environmental Science, University of Vienna, Austria.

* equal contribution.

✉email: ascel.samba@univ-poitiers.fr

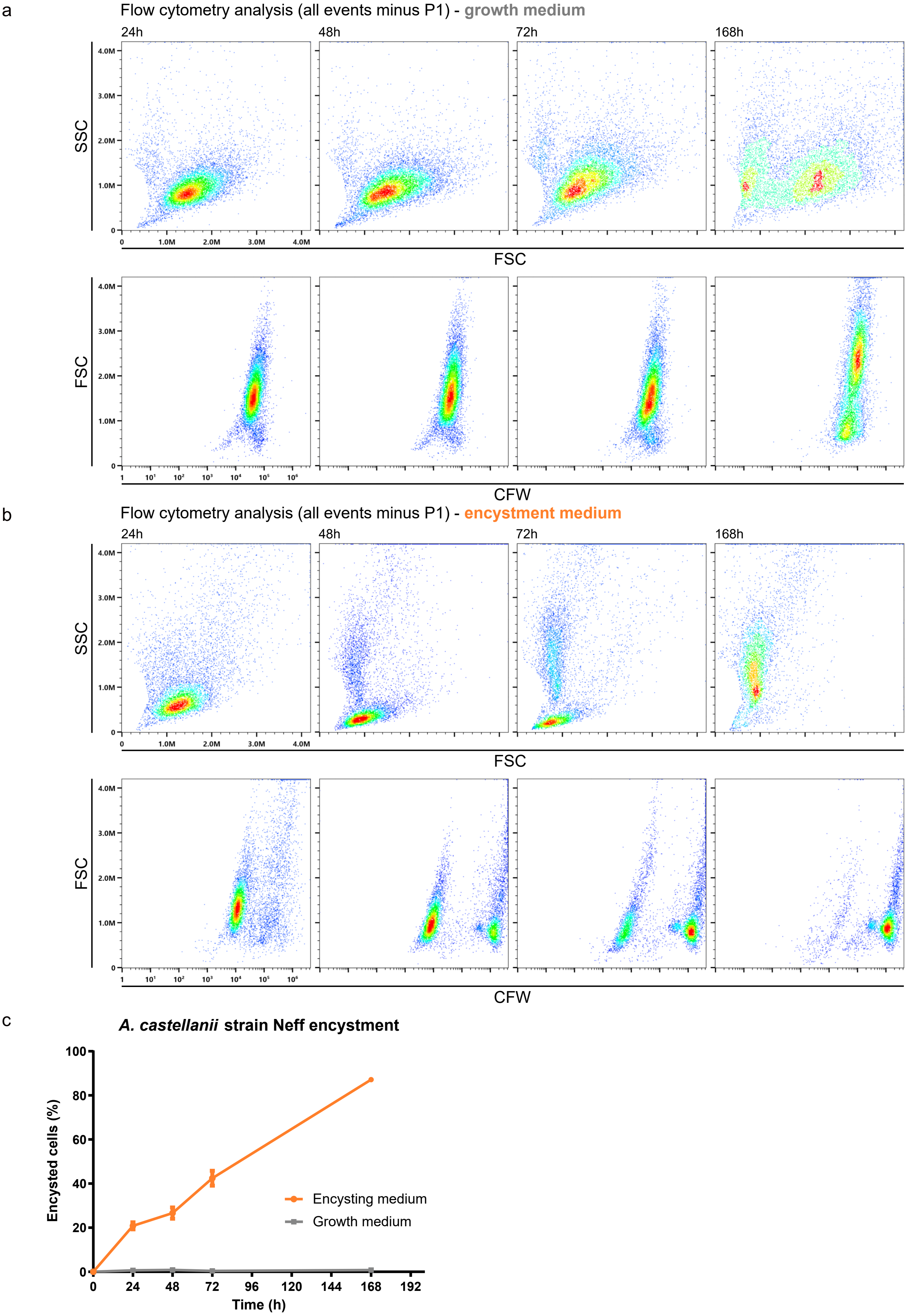

Supplementary Figure 1: Encystment of *A. castellanii* strain Neff. a-b) Characterization of A. castellanii strain Neff cells at 24, 48, 72 and 196h after growth medium renewal **(a)**, or incubation within the encysting medium **(b)**, by flow cytometry (gating strategy shown in Supplementary Fig. 8). First rows of each panel contain FSC vs. SSC dot plots, and second row contain FSC vs. fluorescence intensity dot plots, as all cells were stained with calcofluor-white (CFW) prior to flow cytometry analysis. Data are representative of three independent experiments. **c)** Plot representing the percentage of *A. castellanii* strain Neff cysts quantified by flow cytometry, at 0, 24, 48, 72, and 192h after medium renewal or encystment induction (grey and orange, respectively). n = 3; error bars = SEM.

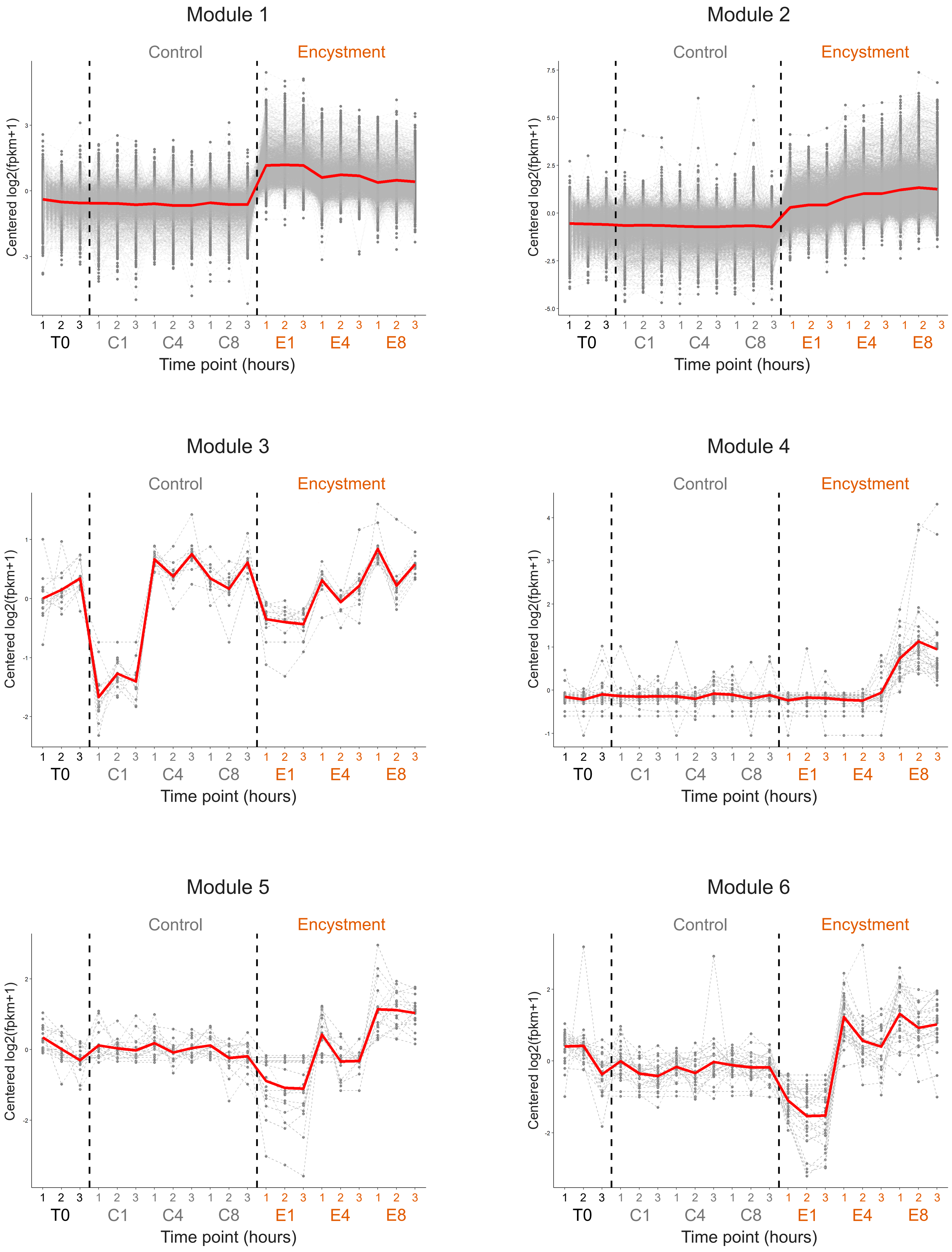

Supplementary Figure 2 - 1/4 : Regulated transcripts modules. The ordinate axis represents each transcript's set of expression values centered by subtracting the median (centered log2(fpkm+1)). Transcripts of each module are plotted as grey lines across all the samples of the data set (x-axis). “E” = encystment condition; “C” = control. The red lines represent the mean expression of each transcript.

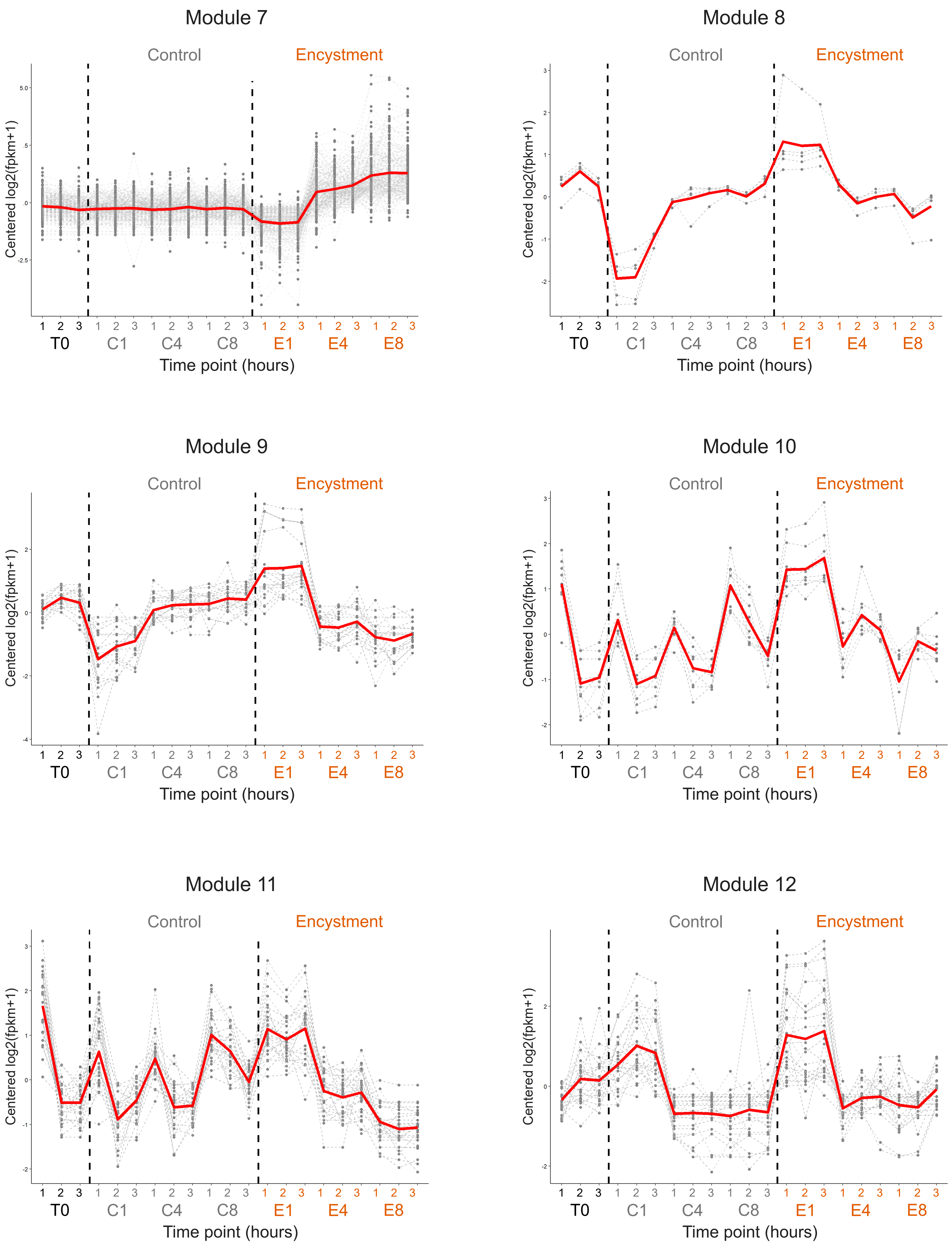

Supplementary Figure 2 - 2/4 : Regulated transcripts modules. The ordinate axis represents each transcript's set of expression values centered by subtracting the median (centered log2(fpkm+1)). Transcripts of each module are plotted as grey lines across all the samples of the data set (x-axis). “E” = encystment condition; “C” = control. The red lines represent the mean expression of each transcript.

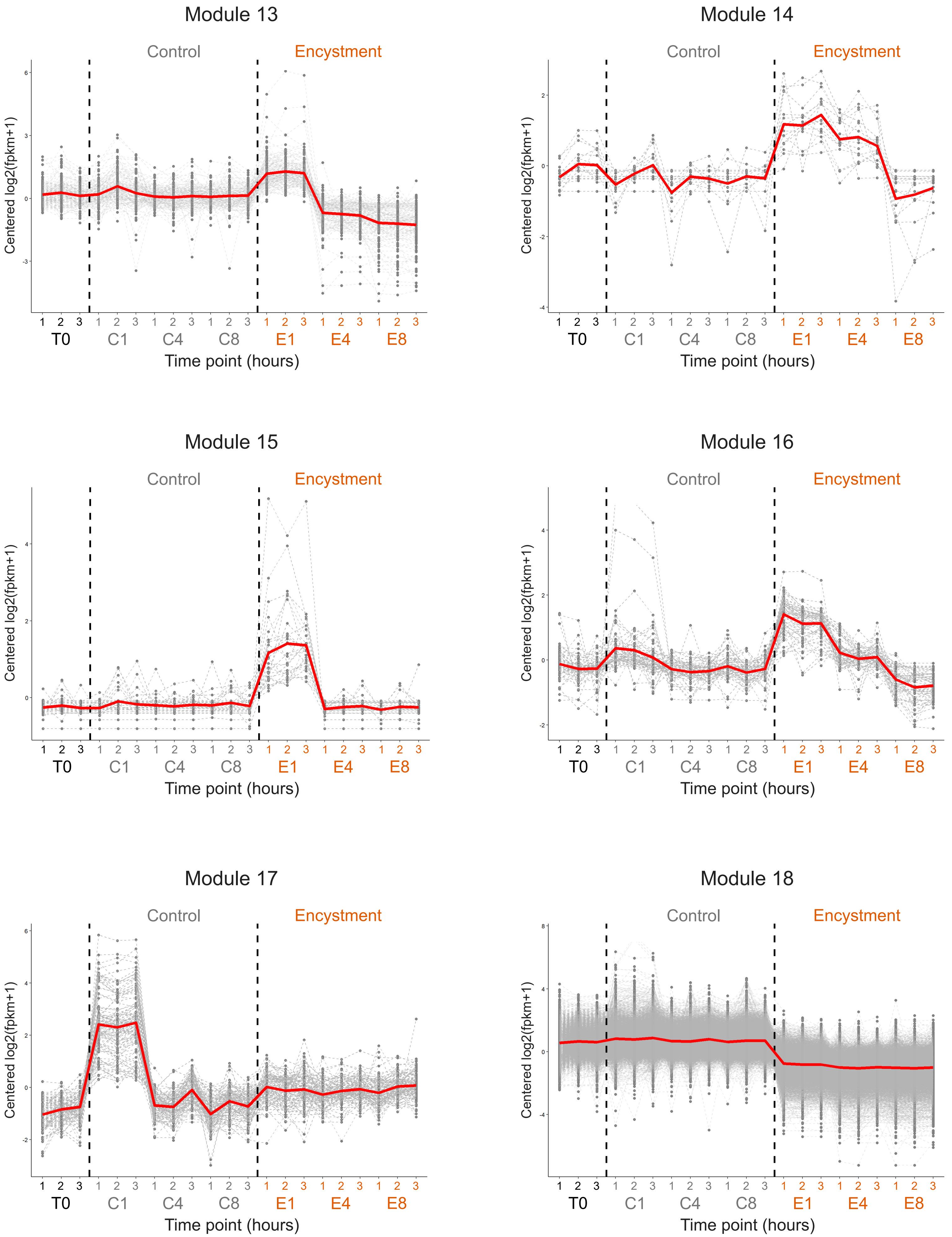

Supplementary Figure 2 - 3/4 : Regulated transcripts modules. The ordinate axis represents each transcript's set of expression values centered by subtracting the median (centered log2(fpkm+1)). Transcripts of each module are plotted as grey lines across all the samples of the data set (x-axis). “E” = encystment condition; “C” = control. The red lines represent the mean expression of each transcript.

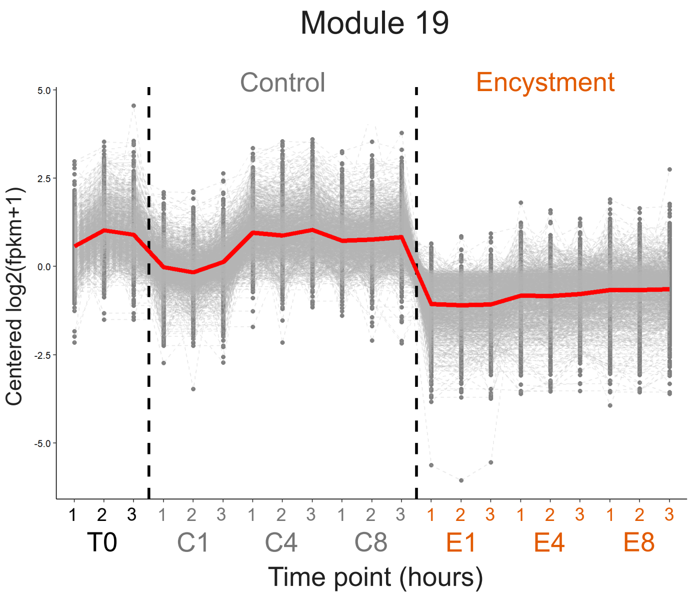

Supplementary Figure 2 - 4/4 : Regulated transcripts modules. The ordinate axis represents each transcript's set of expression values centered by subtracting the median (centered log2(fpkm+1)). Transcripts of each module are plotted as grey lines across all the samples of the data set (x-axis). “E” = encystment condition; “C” = control. The red lines represent the mean expression of each transcript.

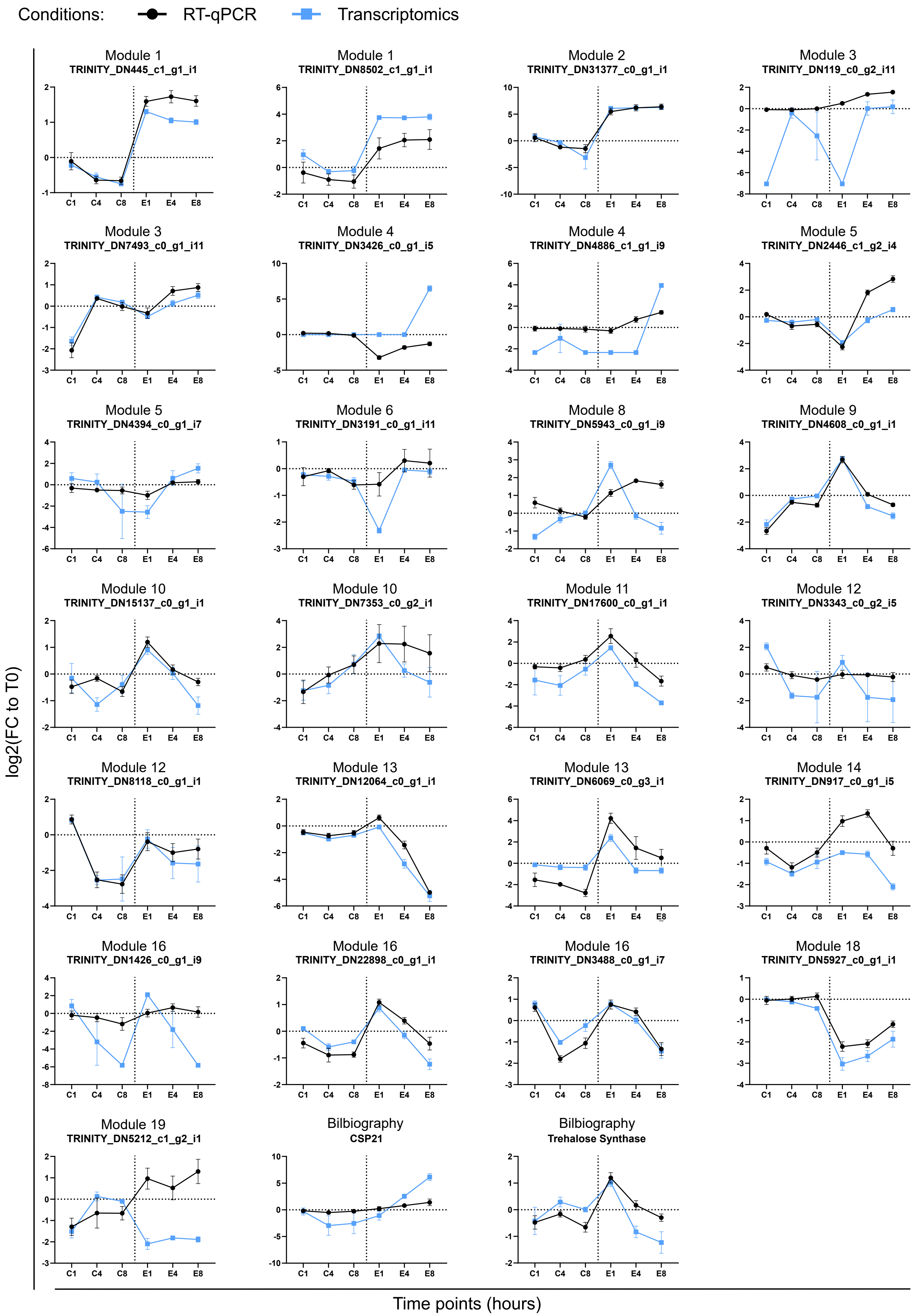

Supplementary Figure 3: RT-qPCR validation of 28 individual transcripts. 28 transcripts from the RNA-seq data were selected for RT-qPCR analysis in the same conditions as the ones utilized for generating the transcriptome. Each plot shows the RNA-seq (blue) and RT-qPCR (black) mean values of log2-transformed fold changes relative to the control (0h). For RT-qPCR, n = 6 independent experiments; for RNA-seq, n = 3 independent experiments; error bars = SEM.

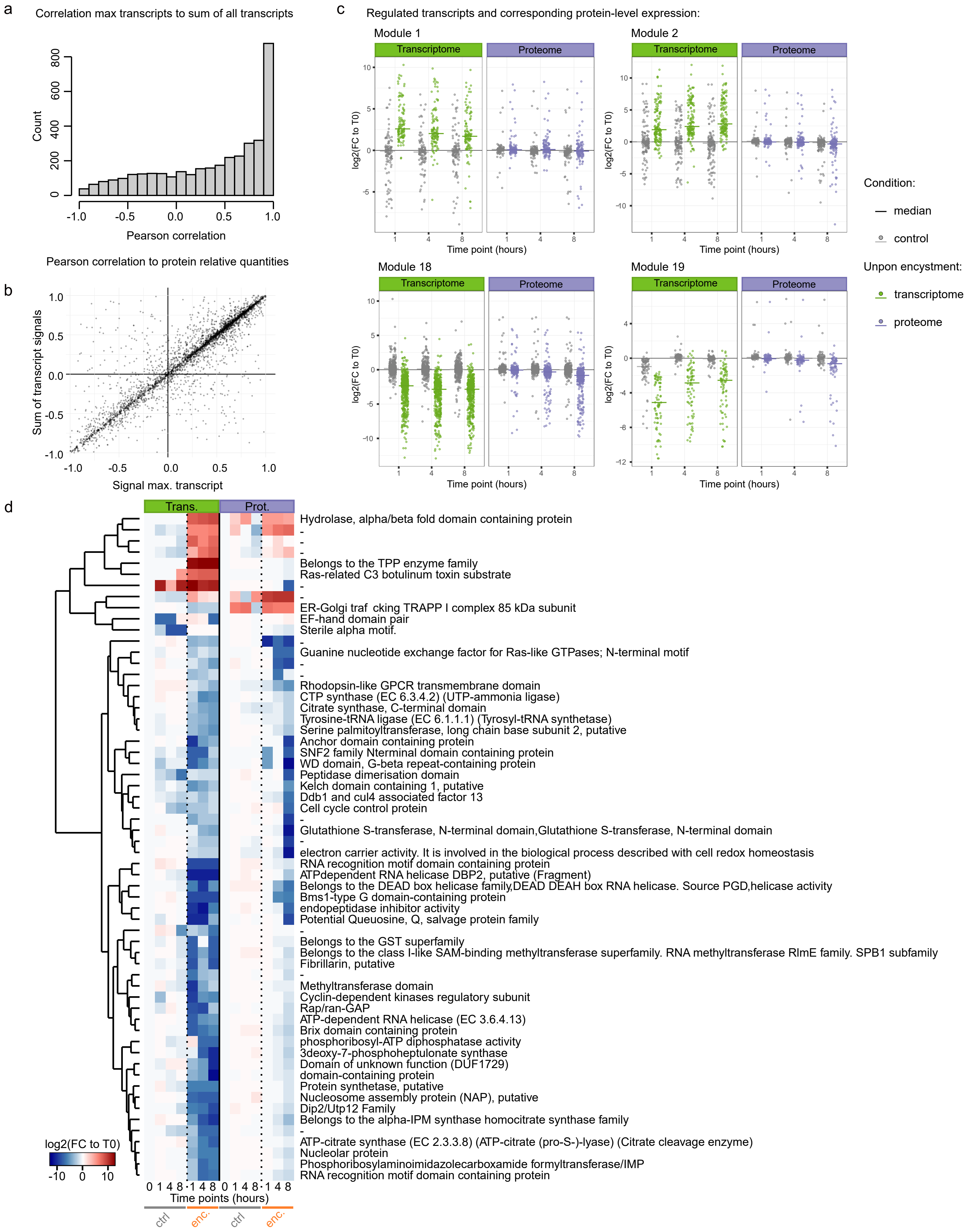

Supplementary Figure 4: Integration of the transcriptome and proteome data sets. a) Histogram of Pearson correlations of protein relative quantities calculated based on the transcript with maximal sum of signal or the sum of all transcripts corresponding to each protein group. **b)** Scatter plot of the Pearson correlations obtained between protein group and matching transcript relative quantities when using the transcript with highest signal (x-axis) or the sum of all matching transcripts (y-axis). **c)** Comparison of transcript- and protein-level regulation. For each transcript module, the log2-transformed fold changes relative to T0 of transcripts matched to protein groups were plotted next to the corresponding protein groups at the three time points (x-axis) in the control (grey) and encystment condition (transcripts and protein groups are in green and purple, respectively). Median values are presented by a horizontal bar. **d)** Heatmap presenting the 60 top hits regulated at both transcript- and protein-level. Labels on the right are based on Uniprot description or eggNOG-mapper output (if no Uniprot description available). "-" indicates that no functional information was retrieved for the given protein sequence.

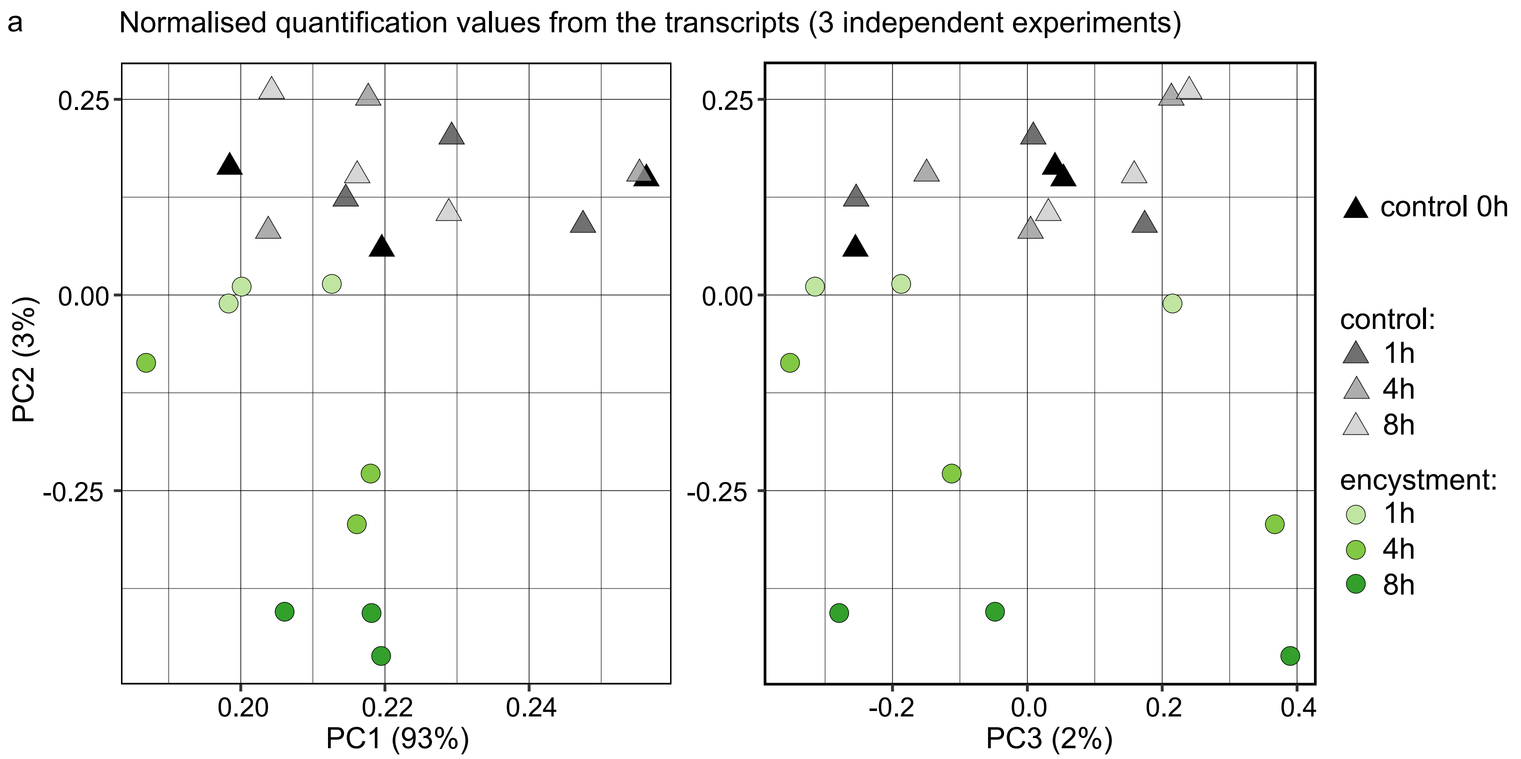

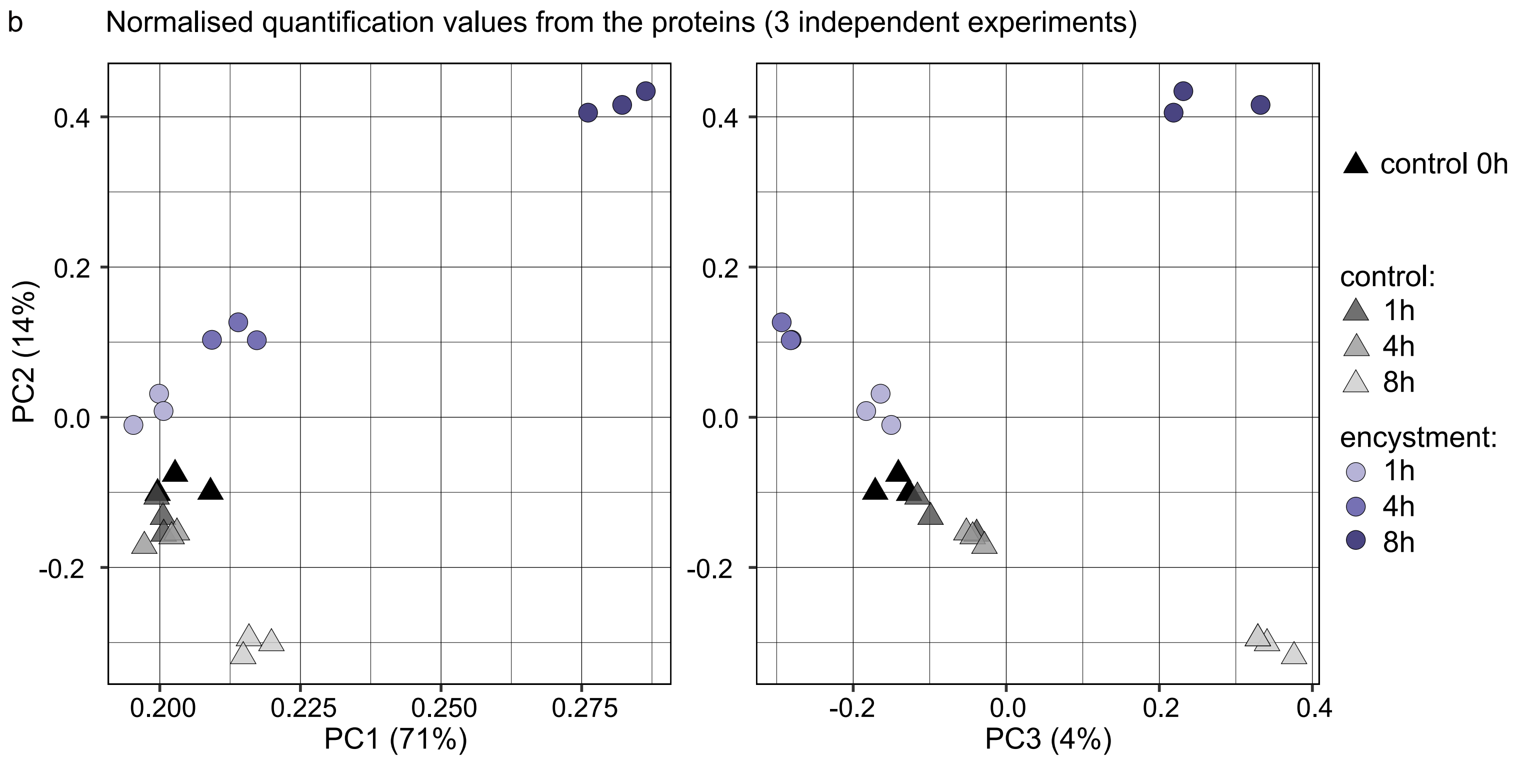

Supplementary Figure 5: Principal Component Analysis (PCA) of the transcriptome and proteome. PCA of the relative quantities in the transcriptome (a) and the proteome (b) before statistical analysis. Each biological replicate is represented by a point, conditions and time points are color-coded. In (b), PCA were plotted after replacement of missing values as described in the methods section of the manuscript.

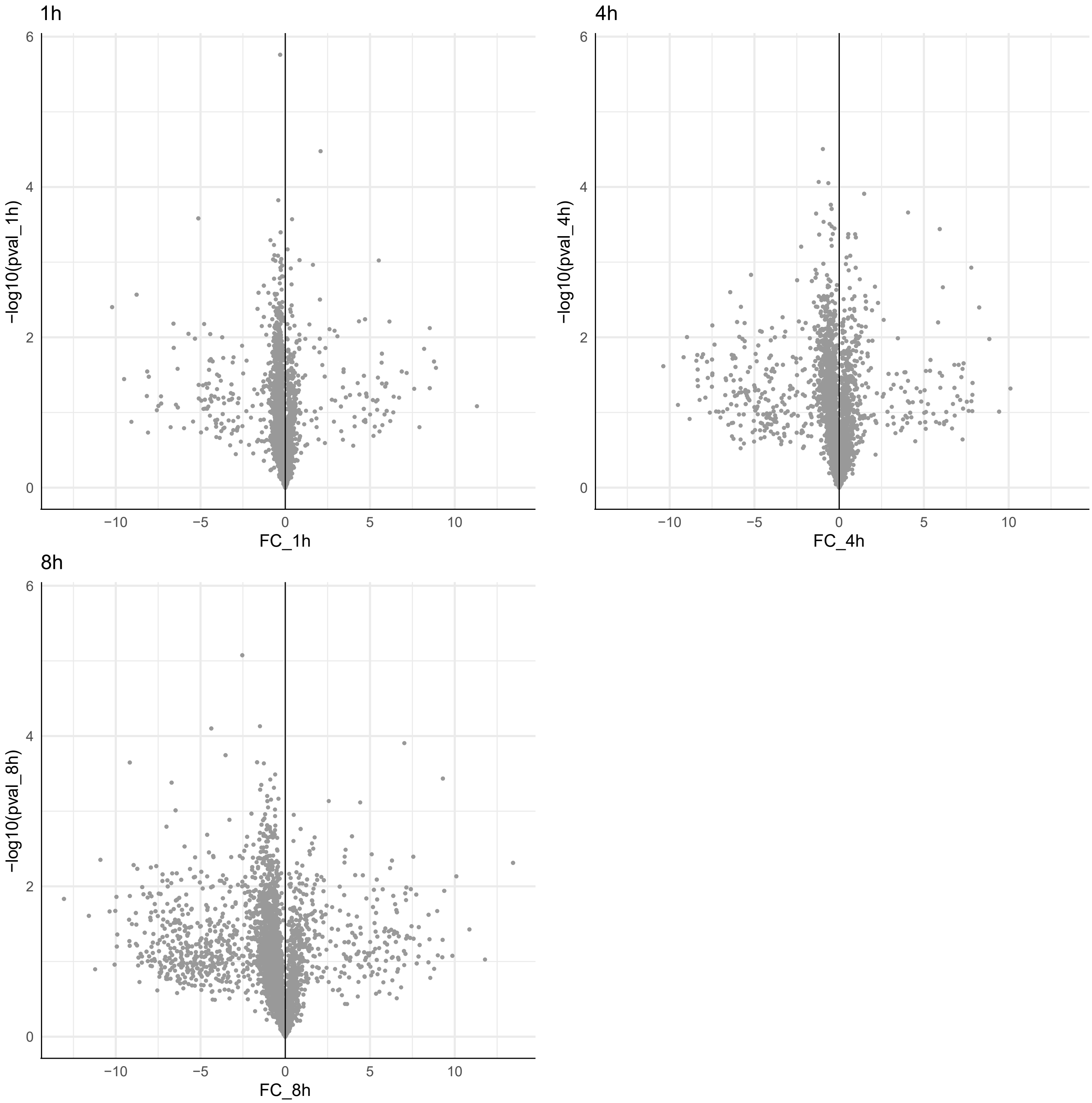

Supplementary Figure 6: Volcano plots highlighting protein regulation reproducibility at different time point. -log10(p-values) from the two-sided paired t-test between encystment and control condition was plotted as a function of the log2-tranformed fold change for each time point.

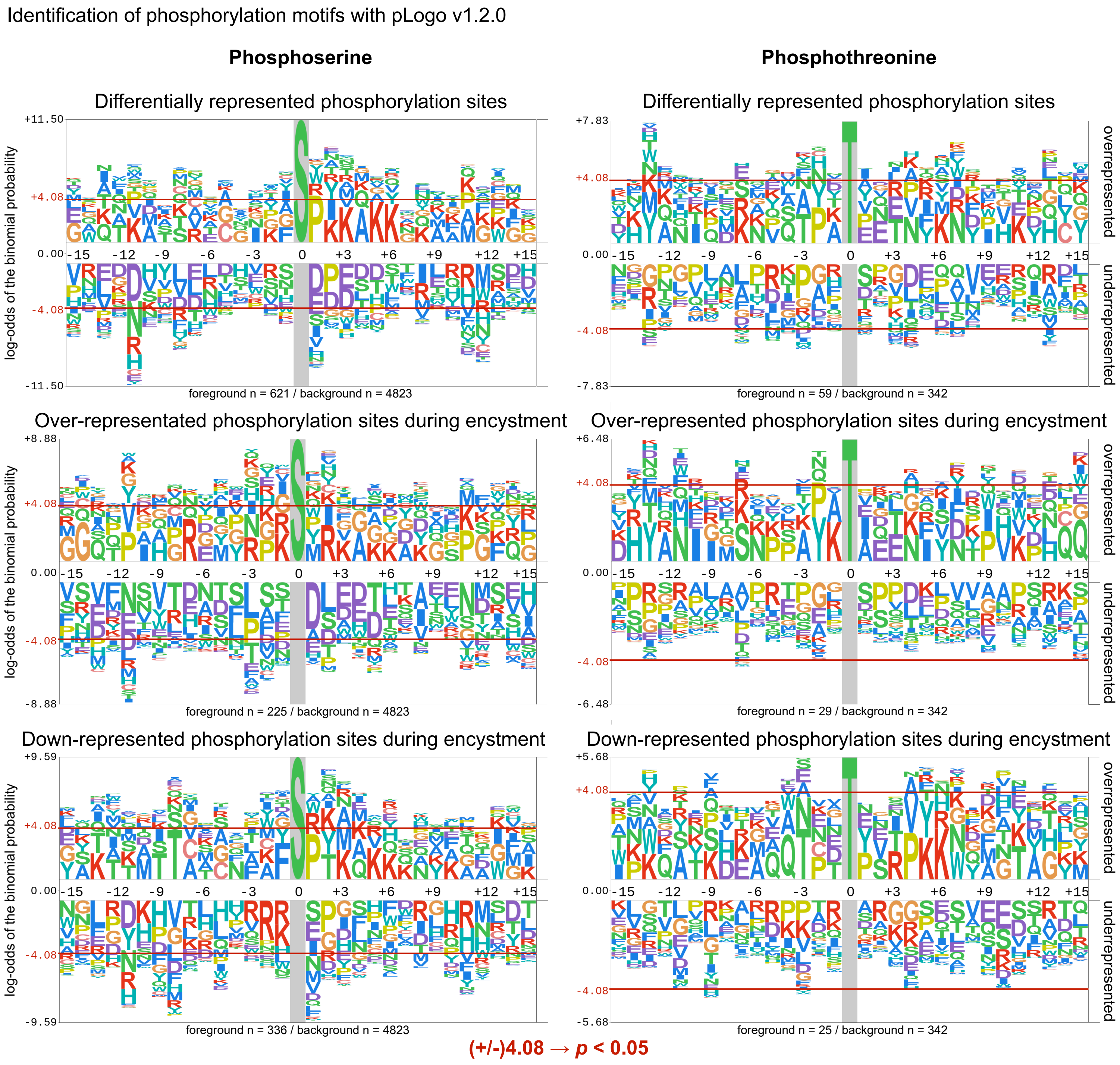

Supplementary Figure 7: Identification of encystment-specific phosphorylation motifs with pLogo v1.2.0 (plogo.uconn.edu). Amino-acid sequences surrounding regulated phosphosites (-15/+15 residues around the phosphorylated serine or threonine) are represented by letters that are scaled to the statistical significance of over- or under-representation (positive and negative log odds of the binomial probability, respectively). The foreground used was either composed of sequences from all phosphosites identified as regulated in tested condition (top panel), from phosphosites over-represented during encystment (middle panel) and from phosphosites under-represented during encystment (bottom panel). The background used was composed of all phosphorylated sequences identified in the phosphoproteome: only phophoserine or phosphothreonine. Red line shows the threshold for a statistical significance difference in representation with p = 0.05. The significance value is evaluated using the binomial probability of residue frequencies, as described by O'Shea *et al.*[32]

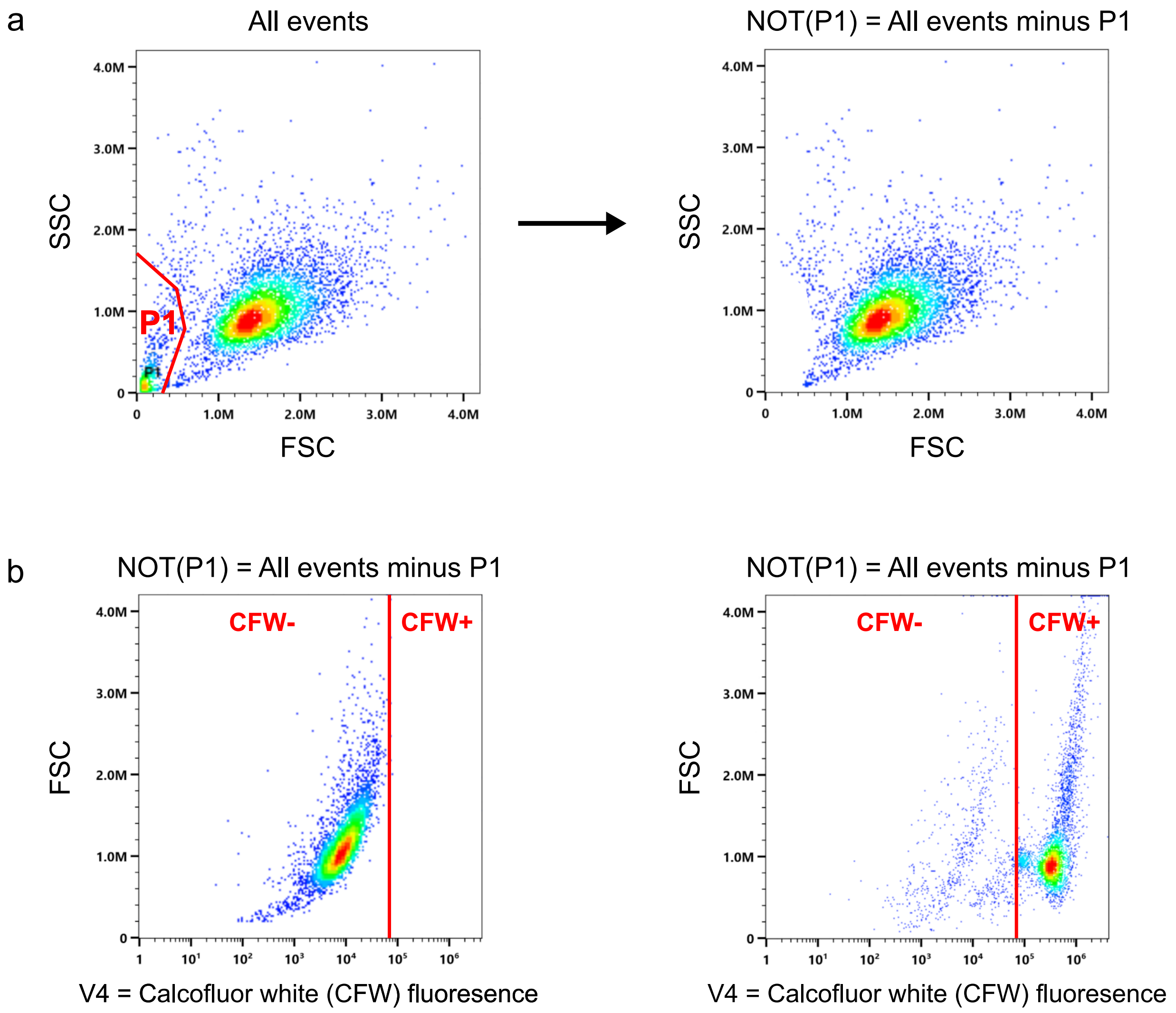

Supplementary Figure 8: Gating strategy used for flow cytometry analysis. a) For each experiment, we used the FSC/SSC gating on all events to remove debris by gating them (gate "P1"), and we then analyzed the samples ("NOT(P1)" = all events minus P1). **b)** Boundaries between "positive" (CFW+) and "negative" (CFW-) staining cell populations were defined with samples that did not receive the staining agent (calcofluor white). The same threshold was then applied to all samples.
