# Supplementary material for: A time-resolved multi-omics atlas of Acanthamoeba castellanii encystment
Source: Nat Commun. 2022 Jul 14;13:4104. doi: 10.1038/s41467-022-31832-0 (PMC9283445; doi:10.1038/s41467-022-31832-0)
Supplement: Supplementary file 3 — Description of Additional Supplementary Information [file 41467_2022_31832_MOESM3_ESM.docx]

**Description of Additional Supplementary Information**

- Title: **Supplementary Data 1**
- Description: **Quantitative transcriptomic data corresponding to the regulated transcripts and corresponding modules.**
- Title: **Supplementary Data 2**
- Description: **Gene Ontology term enrichment results at transcriptome, proteome and phosphoproteome level.** GO term enrichments were performed using a hypergeometric test with the full transcriptome or the full proteome as background for modules or proteome/phospho enrichment, respectively. Genes from significant child-categories were removed with a FWER threshold of 0.1 (R package GOfuncR).
- Title: **Supplementary Data 3**
- Description: **Functional annotation of transcripts using eggNOG-mapper.**
- Title: **Supplementary Data 4**
- Description: **Protein-centric analysis of *A. castellanii* encystment.**
- Title: **Supplementary Data 5**
- Description: **Phosphoproteomic analysis of *A. castellanii* encystment.**
- Title: **Supplementary Data 6**
- Description: **Phosphorylation motif analysis using rmotifx R package.**
- Title: **Supplementary Data 7**
- Description: **List of primers used in this study.**
