# Supplementary material for: A time-resolved multi-omics atlas of Acanthamoeba castellanii encystment
Source: Nat Commun. 2022 Jul 14;13:4104. doi: 10.1038/s41467-022-31832-0 (PMC9283445; doi:10.1038/s41467-022-31832-0)
Supplement: Supplementary file 11 — Reporting Summary [file 41467_2022_31832_MOESM11_ESM.pdf]

nature portfolio

Corresponding author(s): Ascel Samba-Louaka

Last updated by author(s): Jun 13, 2022

Reporting Summary

Nature Portfolio wishes to improve the reproducibility of the work that we publish. This form provides structure for consistency and transparency in reporting. For further information on Nature Portfolio policies, see our Editorial Policies and the Editorial Policy Checklist.

Statistics

For all statistical analyses, confirm that the following items are present in the figure legend, table legend, main text, or Methods section.

n/a	Confirmed	
☐	☒	The exact sample size (n) for each experimental group/condition, given as a discrete number and unit of measurement
☐	☒	A statement on whether measurements were taken from distinct samples or whether the same sample was measured repeatedly
☐	☒	The statistical test(s) used AND whether they are one- or two-sided<br>*Only common tests should be described solely by name; describe more complex techniques in the Methods section.*
☒	☐	A description of all covariates tested
☐	☒	A description of any assumptions or corrections, such as tests of normality and adjustment for multiple comparisons
☐	☒	A full description of the statistical parameters including central tendency (e.g. means) or other basic estimates (e.g. regression coefficient) AND variation (e.g. standard deviation) or associated estimates of uncertainty (e.g. confidence intervals)
☐	☒	For null hypothesis testing, the test statistic (e.g. F, t, r) with confidence intervals, effect sizes, degrees of freedom and P value noted<br>*Give P values as exact values whenever suitable.*
☒	☐	For Bayesian analysis, information on the choice of priors and Markov chain Monte Carlo settings
☐	☒	For hierarchical and complex designs, identification of the appropriate level for tests and full reporting of outcomes
☐	☒	Estimates of effect sizes (e.g. Cohen's d, Pearson's r), indicating how they were calculated

Our web collection on statistics for biologists contains articles on many of the points above.

Software and code

Policy information about availability of computer code

Data collection	Mass spectra were acquired with the XCalibur 2.2 software (Thermo Fisher Scientific, Germany).<br>Flow cytometry data were acquired with the SpectroFlo® 2.2 software (Cytek Biosciences, USA).
Data analysis	SpectroFlo® v2.2 at https://cytekbio.com/pages/spectro-flo<br>MaxQuant (with the Andromeda search engine) v1.5.3.8 at https://www.maxquant.org/<br>eggNOG-mapper v2.0 with eggNOG v5.0 database at http://eggnog-mapper.embl.de<br>Primer-BLAST (based Primer3 v4.1.0) at https://www.ncbi.nlm.nih.gov/tools/primer-blast/<br>Trinity v2.6.6 at https://github.com/trinityrnaseq/trinityrnaseq/releases<br>Assemblathon v2 at https://github.com/KorfLab/Assemblathon<br>Bowtie2 v2.2.1 at http://bowtie-bio.sourceforge.net/bowtie2/index.shtml<br>TrimGalore v0.6.1 at https://www.bioinformatics.babraham.ac.uk/projects/trim_galore/<br>Blobtools v1.1 at https://github.com/DRL/blobtools<br>Rcorrector v1.0.3 at https://github.com/mourisl/Rcorrector<br>R v4.0.3 at https://www.r-project.org/<br>DESeq2 v1.26.0 at https://anaconda.org/bioconda/bioconductor-deseq2<br><br>R packages:<br>imp4p v0.9 at https://cran.rstudio.com/web/packages/imp4p/index.html<br>imputeLCMD v2.0 at https://cran.rstudio.com/web/packages/imputeLCMD/index.html<br>GOfuncR v1.10.0 at https://www.bioconductor.org/packages/release/bioc/html/GOfuncR.html<br>WGCNA v1.69 at https://cran.r-project.org/web/packages/WGCNA/index.html<br>DESeq2 V1.35.0 at http://www.bioconductor.org/packages/release/bioc/html/DESeq2.html

March 2021

rmotifx V1.0 at https://github.com/omarwagih/rmotifx

Transcriptomic data were processed using a FAIR workflow built specifically for this project. This pipeline is publicly accessible at https://github.com/ifremer-bioinformatics/FLORA.

The computational analysis of the proteome, the phosphoproteome and their integration with the transcriptome were done with R v4.0.3, and all the custom script designed during this study are available on Zenodo with the DOI 10.5281/zenodo.5869928.

For manuscripts utilizing custom algorithms or software that are central to the research but not yet described in published literature, software must be made available to editors and reviewers. We strongly encourage code deposition in a community repository (e.g. GitHub). See the Nature Portfolio guidelines for submitting code & software for further information.

Data

Policy information about availability of data

All manuscripts must include a data availability statement. This statement should provide the following information, where applicable:

- Accession codes, unique identifiers, or web links for publicly available datasets
- A description of any restrictions on data availability
- For clinical datasets or third party data, please ensure that the statement adheres to our policy

All transcriptomic raw data generated in this study have been deposited in the NCBI SRA database, under the BioProject database identifier PRJNA794325.
The MS proteomics data generated in this study have been deposited to the ProteomeXchange Consortium via the PRIDE partner repository, under the project accession number PXD031788.
FASTA used for the computational analysis and the protein identification and quantification used in this study are available on Zenodo with the DOI 10.5281/zenodo.5869928.
The raw data for all figures generated in this study are included in the supplementary data files or in the source data file.
The following genomes were used in this study: Acanthamoeba castellanii strain Neff [GCA_000313135.1, BioProject PRJNA66753], Acanthamoeba castellanii strain Namur [GCA_903821525.1], Acanthamoeba polyphaga strain Linc Ap-1 [GCA_001567625.1], Dictyostelium discoideum [GCA_000004695.1], Naegleria gruberi [GCA_000004985.1].

Field-specific reporting

Please select the one below that is the best fit for your research. If you are not sure, read the appropriate sections before making your selection.

☒ Life sciences ☐ Behavioural & social sciences ☐ Ecological, evolutionary & environmental sciences

For a reference copy of the document with all sections, see nature.com/documents/nr-reporting-summary-flat.pdf

Life sciences study design

All studies must disclose on these points even when the disclosure is negative.

Sample size	No statistical methods were used to predetermine sample sizes. They were determined based on common practice in the field, and are in line with previous publications.
Data exclusions	No samples were excluded from this study.
Replication	The number of replicates based on what was technically/practically/financially possible and what is currently done in the field. For transcriptomic, proteomic and phosphoproteomic and encystment assay, all experiments were performed in triplicate. RT-qPCR validation and phosphastase assays were carried out with 6 series of independant samples.
Randomization	For each experiment, after initial culture, cells were randomly assigned in either the control group (growth medium) or the test group (encysting medium).
Blinding	The investigators were not blinded as proper controls were included during experiment design.

Reporting for specific materials, systems and methods

We require information from authors about some types of materials, experimental systems and methods used in many studies. Here, indicate whether each material, system or method listed is relevant to your study. If you are not sure if a list item applies to your research, read the appropriate section before selecting a response.

Materials & experimental systems

n/a	Involved in the study
☒	☐ Antibodies
☐	☒ Eukaryotic cell lines
☒	☐ Palaeontology and archaeology
☒	☐ Animals and other organisms
☒	☐ Human research participants
☒	☐ Clinical data
☒	☐ Dual use research of concern

Methods

n/a	Involved in the study
☒	☐ ChIP-seq
☐	☒ Flow cytometry
☒	☐ MRI-based neuroimaging

Eukaryotic cell lines

Policy information about cell lines

Cell line source(s)	Acanthamoeba castellanii strain Neff, source : ATCC (30010)
Authentication	Certificate of analysis was provided by the ATCC organization.
Mycoplasma contamination	The cell lines were tested for mycoplasma contamination by the supplier.
Commonly misidentified lines (See ICLAC register)	n/a

Flow Cytometry

Plots

Confirm that:

☒ The axis labels state the marker and fluorochrome used (e.g. CD4-FITC).

☒ The axis scales are clearly visible. Include numbers along axes only for bottom left plot of group (a 'group' is an analysis of identical markers).

☒ All plots are contour plots with outliers or pseudocolor plots.

☒ A numerical value for number of cells or percentage (with statistics) is provided.

Methodology

Sample preparation	A. castellanii trophozoites were seeded into 6 wells microplates at a density of 2.105 cells in 3 mL of PYG medium and incubated at 30 °C overnight. Afterward, for growth condition, PYG medium was replaced by 3 mL of fresh PYG medium. For encysting condition, cells were rinsed twice with 3 mL of Page's Amoeba Saline (PAS) buffer (0.1% sodium citrate dihydrate, 0.4 mM CaCl2, 4 mM MgSO4, 0.05 mM Fe(NH4)2(SO4)2 6H2O, 2.5 mM K2HPO3, 2.5 mM NaH2PO3, pH 6.5) and then 3 mL of encysting buffer (0.1 M KCl, 0.4 mM CaCl2, 1 mM NaHCO3, 8 mM MgSO4, and 20 mM 2-amino-2-methyl-1,3-propanediol, pH 8.8) were added to the wells. Cells were incubated at 30 °C for 24, 48, 72 or 168 h to evaluate A. castellanii encystment ability. For growth condition, supernatant was eliminated and 3 mL of PBS containing EDTA (137 mM NaCl, 2.7 mM KCl, 10 mM NaHPO4, 1.8 mM KH2PO4, 2 mM EDTA, pH 7.4) were added to the well. For encysting condition, wells received EDTA to reach a final concentration of 2 mM. For both condition, cells were detached using cell-scrapers and suspensions were transferred in 5 mL, 75 x 12 mm, flow cytometry tubes after filtration on 70 µm cell strainers. Cells were stained using 10 µL of calcofluor white reagent that allow trophozoites and cysts distinction. Samples were analyzed on a Cytek® Aurora (Cytek Biosciences, USA)
Instrument	Cytek® Aurora, Cytek Biosciences, USA
Software	SpectroFlo® (Cytek Biosciences, USA) version 2.2 was used to collect and analyze the flow cytometry data.
Cell population abundance	Cell sorting was not employed
Gating strategy	For each experiment, we used the FSC/SSC gating on all events to remove debris by gating them (gate "P1"), and we then analyzed the samples ("NOT(P1)" = all events minus P1).<br>Boundaries between "positive" and "negative" staining cell populations were defined with samples that did not receive the staining agent (calcofluor white). The same threshold was then applied to all samples.<br>Gating strategy is described in Supplementary Fig. 8.

☒ Tick this box to confirm that a figure exemplifying the gating strategy is provided in the Supplementary Information.
